# Social Media Literacy and Health Information-Seeking Behavior Among School Students and Educators in India: A Needs Assessment Under Project Info-Suraksha

**DOI:** 10.7759/cureus.117376

**Published:** 2026-10-03

**Authors:** Rajni Kant, Bhavna B Mukhopadhyay, Nancepreet Kaur, Swapnil Jain, Saritha Nair, Enna Dogra Gupta, Shireen Fatma, Nhu Nguyen Tran Minh, Sophia Lonappan, Neha Srivastava

**Affiliations:** 1 Health Policy and Communication, Indian Council of Medical Research, New Delhi, IND; 2 Health Policy and Communication, Voluntary Health Association of India, New Delhi, IND; 3 Health Promotion and Noncommunicable Diseases, Voluntary Health Association of India, New Delhi, IND; 4 Community Medicine, Indian Council of Medical Research-National Institute for Research in Digital Health, New Delhi, IND; 5 Health Sciences, Voluntary Health Association of India, New Delhi, IND; 6 Health Sciences, World Health Organization (WHO) India, New Delhi, IND; 7 Public Health and Health Risk Communication, World Health Organization (WHO) India, New Delhi, IND; 8 Viral Immunology and Vaccine Research, Indian Council of Medical Research-National Institute of Health Research, Gorakhpur, IND

**Keywords:** disinformation, education intervention, fake news, infodemic, misinformation, need assessment, online health information, social media literacy, training of teachers

## Abstract

Purpose

The increasing use of social media has made social media literacy vital for promoting safe, responsible, and informed online engagement. This study aimed to evaluate the knowledge, behaviors, and practices of school students and educators regarding social media usage and online health information seeking. The central research question focused on understanding how effectively students and educators navigate digital spaces and assess the credibility of online health information.

Methods

A cross-sectional mixed-methods needs assessment study was conducted across five Indian cities, encompassing 15 schools per city representing public, semi-private, and private institutions. The total sample included 2,331 students (grades 9-12) and 373 educators. A mixed-method approach, integrating quantitative surveys and qualitative interviews, was used.

Results

Internet access was reported by 97.5% of students and all educators. Among students, WhatsApp (Meta Platforms, Inc., Menlo Park, California, United States) (88.6%), YouTube (Alphabet Inc., Mountain View, California, United States) (85.2%), and Instagram (Meta Platforms, Inc.) (64%) were the most used platforms. Both social media (60.5%) and traditional media (58.5%) were primary sources of health information. While 88.4% of educators and 77.7% of students searched for health news online mainly via WhatsApp, YouTube, Instagram, and Facebook (Meta Platforms, Inc.), 33.2% of educators were unsure about information accuracy, but 55% still shared it. Only 4-6% of participants had received prior training on identifying misinformation.

Conclusion

The findings underscore the urgent need for structured capacity-building programs that enhance social media literacy, promote critical evaluation of online health content, and foster digital citizenship to mitigate misinformation and encourage responsible online behavior.

## Introduction

Over the recent years, the pervasive influence of social media platforms has significantly reshaped the landscape of information dissemination and communication. According to the Telecom Regulatory Authority of India (TRAI), there were 1,092.79 million internet users in India by March 2026 [1]. The reliance on social media/internet has increased over the years for all sorts of information, including health, facilitated by the widespread use of mobile devices and affordable internet connectivity. However, alongside the myriad benefits of this digital age, there exists a formidable challenge: the rise of an “infodemic”. An infodemic is defined as an abundance of information, both false and misleading, along with correct and useful information, in digital and physical environments during a disease outbreak, as seen during the COVID-19 pandemic [2]. Amidst this deluge of information, an infodemic poses a significant threat to societal well-being, public health, and democratic discourse on disease control, as it jeopardizes healthcare measures.

School students and educators stand at the forefront of navigating this complex digital landscape, as schools serve as hubs for reaching a broad ecosystem of young and adolescent children. Through collaboration with parents and communities, schools can wield significant influence as a catalyst in fostering a healthy digital ecosystem, both within and beyond their premises [3]. Therefore, their ability to critically evaluate online content is of paramount importance. 

Understanding the imperative for social media literacy, the World Health Organization (WHO) Country Office in India (WHO India) and the Indian Council of Medical Research (ICMR), in collaboration with Voluntary Health Association of India (VHAI) conducted a unique project named "Project Info-Suraksha" (Info-Safety), the motto fo which is "Learn -Verify - Share to Empower School Communities on Responsible Use of Social Media and Infodemic Management through Capacity Building” in five states of India: Uttar Pradesh (UP), Odisha, Gujarat, Assam, and Andhra Pradesh from May to November, 2023 [4]. Social media literacy encompasses the knowledge, skills, and capacity to access, comprehend, and critically assess information across various social media platforms. It also involves becoming a responsible digital citizen who uses social media ethically, safely, and responsibly [5].

The primary objective of the study was to conduct a needs assessment among school students and educators in selected schools of India between May and July 2023, focusing on their knowledge, attitudes, and practices related to social media use. The secondary objective was to assess online health-information-seeking behaviour within school settings. The assessment also explored the sources and channels used to access and disseminate health information, as well as the strategies employed by students and educators to identify, evaluate, and respond to fake news and health misinformation.

## Materials and methods

This study was conducted in five states of India from May 4, 2023, to July 30, 2023. The five states of India included in the study were Gujarat, Uttar Pradesh, Assam, Odisha, and Andhra Pradesh. Employing a structured approach, it utilized both quantitative and qualitative methodologies, incorporating closed-ended questionnaires and open-ended group discussions. The needs assessment encompassed school students and educators, ensuring equitable participation from both genders. For students, assent was obtained, along with permission from school authorities, while informed consent was obtained from educators prior to participation.

Eligibility criteria

The study included school students and educators from selected schools in five cities of Andhra Pradesh, Assam, Gujarat, Odisha, and Uttar Pradesh. Students enrolled in grades 9th to 12th and aged approximately 12-16 years were eligible to participate in the study. Both male and female students were included. Educators teaching in the selected schools, irrespective of subject specialization, were also included. Schools selected for participation comprised government, aided/semi-private, and private institutions to ensure representation from diverse educational settings. Participants were required to be present on the day of data collection. Administrative staff, non-teaching personnel, and parents were excluded.

Study tools

Quantitative Assessment

Two sets of questionnaires (see Appendices), comprising closed-ended questions, were developed for educators and students, respectively. The questionnaires covered various topics, including the accessibility of internet-enabled devices, social media usage (types of platforms and frequency of use), types of content accessed online, major sources of health information, knowledge about the accuracy of health information, the presence of fake news online, understanding of misinformation (false or inaccurate information shared unintentionally) and disinformation (false or inaccurate information shared deliberately, intended to mislead), and awareness of reliable health information sources.

Qualitative Assessment

Qualitative assessment was conducted through focus group discussions to gain a deeper understanding and avoid biases that may arise when filling out closed-ended questionnaires. Focus group discussions allow for more nuanced insights, facilitate open communication, and provide a platform for participants to express their thoughts and experiences in greater detail. Two distinct sets of open-ended questions were crafted for students and educators (see Appendices). These questions focused on the usage of social media, commonly used social platforms, total duration spent on social media daily, types of information accessed online, common types of health information accessed online, understanding the presence of fake news, encounters with fake news online, differentiating between misinformation and disinformation, knowledge about reliable sources of health information, and reporting of fake news.

Translation of the Study Tools

To facilitate a more comprehensive assessment process and enhance understanding, the assessment questionnaires were translated into the local languages, including Hindi, Oriya, Assamese, Telugu, and Gujarati. This effort helped to promote inclusivity and accuracy in data collection.

Study sites and target population

The study was carried out in 75 schools across five cities spanning five states of India, representing diverse regions of the country: (i) Visakhapatnam from Andhra Pradesh, (ii) Guwahati from Assam, (iii) Bhubaneswar from Odisha, (iv) Lucknow from Uttar Pradesh, and (v) Ahmedabad from Gujarat. These cities and states were strategically selected to cover the North, East, West, South, and Northeastern regions of India. In each state, 15 schools were chosen, including a mix of government, private, and aided institutions, catering to students from grades 9th to 12th along with educators. The participating schools were identified and selected by the respective State Departments of Education in accordance with the study requirements and local feasibility.

Sample size and sampling technique

The study utilized a stratified random sampling technique to select participants from each school, with stratification by class and gender. The sample size was calculated using a standard statistical formula, $n_0 = \frac{Z^2pq}{e^2}$, and for a finite population, n = $\frac{n_0}{1 + \frac{n_0 - 1}{N}}$, where n0 = initial sample size, Z= Z-score for the confidence level (95% confidence → 1.96), p = expected population proportion (50% → 0.50), e = margin of error (5% → 0.05), n = final sample size, N = population size (7,500 for students; 750 for educators).

Accordingly, 2,331 students and 373 educators were included in the study. For items specifically related to internet and social media use and online health-information-seeking behaviour, analyses were restricted to students who reported internet and/or social media access (n = 2,272).

For qualitative assessment, a total of 150 focus group discussions were conducted across 75 schools. Each school hosted two group discussions: one with a group of 25-30 students and another with a group of six educators. This resulted in 75 group discussions with students and 75 with educators. 

Pilot testing

To ensure the efficacy of the objective and subjective questionnaires, pilot testing was conducted in one school per state for both qualitative and quantitative assessment tools. This phase involved 10 students and two to three educators from each school, aiming to assess participants' understanding of the questionnaire and their ability to provide clear responses. Based on the feedback received during the pilot testing, minor adjustments were made before the full-scale survey. 

Statistical analysis

A mixed-methods approach was employed to ensure a comprehensive analysis of the data by combining both quantitative and qualitative techniques. The study leverages the strengths of each method, ensuring a balanced and thorough examination of the research questions. The collected data were analysed using Microsoft Excel (Microsoft Corporation, Redmond, Washington, United States), which facilitated the efficient organization, visualization, and interpretation of both quantitative and qualitative data.

Data were analysed using descriptive statistical methods. Categorical variables were summarized as frequencies and percentages to describe patterns of access, exposure, perception, confidence, and practices related to health information and misinformation among students and teachers. For indicators involving multiple response options (e.g., understanding of misinformation/disinformation), multiple response analysis was performed, and proportions were calculated based on the total number of respondents for each item. Consequently, percentages for these variables may exceed 100%. State-wise comparisons were conducted to examine variations across geographic regions. Additionally, subgroup analyses were performed by gender (boys vs. girls) and school type (government, aided, and private) to assess disparities in access to internet-enabled devices. No inferential statistical tests were applied, as the study primarily aimed to provide a descriptive overview of patterns and trends. All analyses were conducted using Microsoft Excel, and results are presented in the form of tables and figures for clarity and ease of interpretation.

The qualitative data obtained from the FGD were analyzed using thematic analysis. The analysis involved repeated reading of the FGD transcripts to familiarize the researchers with the data, followed by identification and coding of relevant statements and concepts. Similar codes were grouped into categories, which were subsequently reviewed and organized into broader themes and subthemes aligned with the study objectives. The themes were then interpreted to identify common patterns, perceptions, experiences, and challenges related to social media use and online health-information seeking among students and educators. The qualitative findings were used to complement and provide contextual interpretation of the quantitative findings.

Ethical consideration

Written permissions were secured from the State and District Education Government Departments in all five states. The schools were selected in close consultation with the District Education Departments of the states. Written approvals were granted by the school authorities to conduct the needs assessment. Informed consent was provided by the participating teachers. For student participation, assent was obtained from each student taking part in the survey, and informed consent was given by the school principal. These steps ensured adherence to ethical considerations in data collection. The tools and design underwent approval by both ICMR and WHO prior to implementation in the schools.

## Results

Characteristics of the respondents

Respondents included school students and educators from 75 selected schools representing government, private, and aided institutions across five states in India, covering the northern, western, eastern, northeastern, and southern regions. Student participants were aged 12-16 years (grades 9-12), with a balanced gender distribution, with 1233 girls (52.9%) and 1098 boys (47.1%). Educator respondents were aged 25-60 years, with 244 (65.4%) women and 129 (34.6%) men (Table 1). 

**Table 1:** State-wise distribution of the number of schools, students (by gender), and teachers (by gender)

State	No. of Schools	Students			Teachers		
		Boys, n (%)	Girls, n (%)	Total, n	Male, n (%)	Female, n (%)	Total, n
Gujarat	15	235 (49.4%)	241 (50.6%)	476	31 (42.5%)	42 (57.5%)	73
Uttar Pradesh	15	166 (36.2%)	292 (63.8%)	458	15 (20.0%)	60 (80.0%)	75
Odisha	15	235 (50.2%)	233 (49.8%)	468	28 (35.4%)	51 (64.6%)	79
Assam	15	215 (45.8%)	254 (54.2%)	469	26 (34.7%)	49 (65.3%)	75
Andhra Pradesh	15	247 (53.7%)	213 (46.3%)	460	29 (40.8%)	42 (59.2%)	71
Total	75	1098 (47.1%)	1233 (52.9%)	2331	129 (34.6%)	244 (65.4%)	373

Outcomes of needs assessment survey among students

The survey findings revealed that 2,272 of 2,331 students (97.5%) across all states had access to internet-enabled devices at their homes. Among the states, UP and Odisha reported the highest accessibility, each at 99.1%, indicating near-universal internet availability among students. The minimal variation across states (ranging from 96% to 99%) suggests that internet access among students is largely uniform, with only marginal regional differences (Table 2). As far as the gender-wise distribution of students with internet access is concerned, girls outnumbered boys in almost all the states except Andhra Pradesh. Overall, 1214 (98.5%) girls had access to the internet; for boys, the proportion was slightly less, and 1058 (96.4%) showed accessibility (Table 2).

**Table 2:** State-wise comparison of student’s access to internet-enabled devices by overall access and gender

State	Overall Access, n (%) (N)	Girls, n (%) (N)	Boys, n (%) (N)
Gujarat	463 (97.3%) (N=476)	235 (97.5%) (N=241)	228 (97.0%) (N=235)
Uttar Pradesh	454 (99.1%) (N=458)	291 (99.7%) (N=292)	163 (98.2%) (N=166)
Odisha	464 (99.1%) (N=468)	233 (100%) (N=233)	231 (98.3%) (N=235)
Assam	449 (95.7%) (N=469)	251 (98.8%) (N=254)	198 (92.1%) (N=215)
Andhra Pradesh	442 (96.1%) (N=460)	204 (95.8%) (N=213)	238 (96.4%) (N=247)
Overall	2272 (97.5%) (N=2331)	1214 (98.5%) (N=1233)	1058 (96.4%) (N=1098)

Internet accessibility was consistently high across government, aided, and private schools in all study states, with access levels ranging from 90% to 100%. Nearly all students in Odisha and Uttar Pradesh reported internet access irrespective of school type, while slightly lower access was observed among government school students in Assam (n=170, 90.4%). In the consolidated sample, internet access was reported by 814 (95.7%) students in government schools, 494 (98.2%) in aided schools and 964 (98.7%) in private schools (Table 3).

**Table 3:** Accessibility to internet among students by school category (government, aided, and private) across selected states and consolidated sample

State	Type of School	No. of students interviewed	Students with access to internet, n (%)
Gujarat (N=476)	Government	33	33 (100%)
	Aided	287	280 (97.6%)
	Private	156	150 (96.2%)
Uttar Pradesh (N=458)	Government	120	120 (100%)
	Aided	157	156 (99.4%)
	Private	181	178 (98.3%)
Odisha (N=468)	Government	238	234 (98.3%)
	Aided	29	29 (100%)
	Private	201	201 (100%)
Assam (N=469)	Government	188	170 (90.4%)
	Aided	0	0 (0.0%)
	Private	281	279 (99.3%)
Andhra Pradesh (N=460)	Government	272	257 (94.5%)
	Aided	30	29 (96.7%)
	Private	158	156 (98.7%)
Consolidated (n=2331)	Government	851	814 (95.7%)
	Aided	503	494 (98.2%)
	Private	977	964 (98.7%)

Among 2272 students with internet access, WhatsApp (Meta Platforms, Inc., Menlo Park, California, United States) (n=2014, 88.6%), YouTube (Alphabet Inc., Mountain View, California, United States) (n=1935, 85.2%), and Instagram (Meta Platforms, Inc.) (n=1455, 64.0%) emerged as the most frequently used social media platforms (Figure 1). 

**Figure 1:**
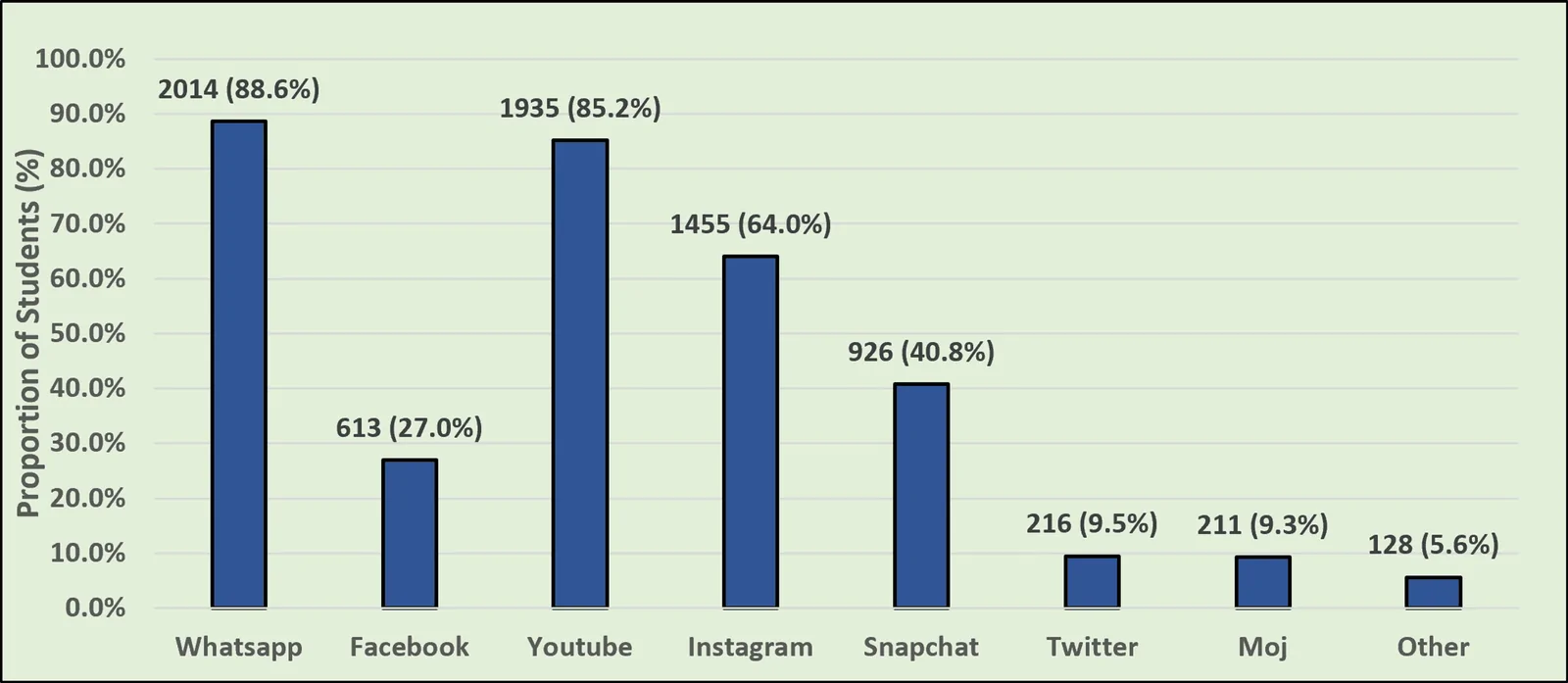
Distribution of social media platform usage among students (N=2272)

A significant portion of students (n=1064, 46.8%) accessed social media multiple times a day, with educational content (n=1222, 53.8%) being the most accessed overall. Entertainment (n=950, 41.8%), interaction with friends (n=878, 38.6%), and access to health news and current events (n=781, 34.4%) were other common uses of social media among students (Table 4, Figure 2). 

**Table 4:** Frequency of social media usage among students across selected states and consolidated sample

States	Students with internet/social media access	Multiple times per day, n (%)	0nce per day, n (%)	Few times per week, n (%)	Rarely or never, n (%)
Gujarat	463	266 (57.5%)	145 (31.3%)	27 (5.8%)	25 (5.4%)
Uttar Pradesh	454	197 (43.4%)	182 (40.1%)	59 (13.0%)	16 (3.5%)
Odisha	464	185 (39.9%)	209 (45.0%)	59 (12.7%)	11 (2.4%)
Assam	449	236 (52.6%)	149 (33.2%)	36 (8.0%)	28 (6.2%)
Andhra	442	180 (40.7%)	175 (39.6%)	65 (14.7%)	22 (5.0%)
Consolidated	2272	1064 (46.8%)	860 (37.9%)	246 (10.8%)	102 (4.5%)

**Figure 2:**
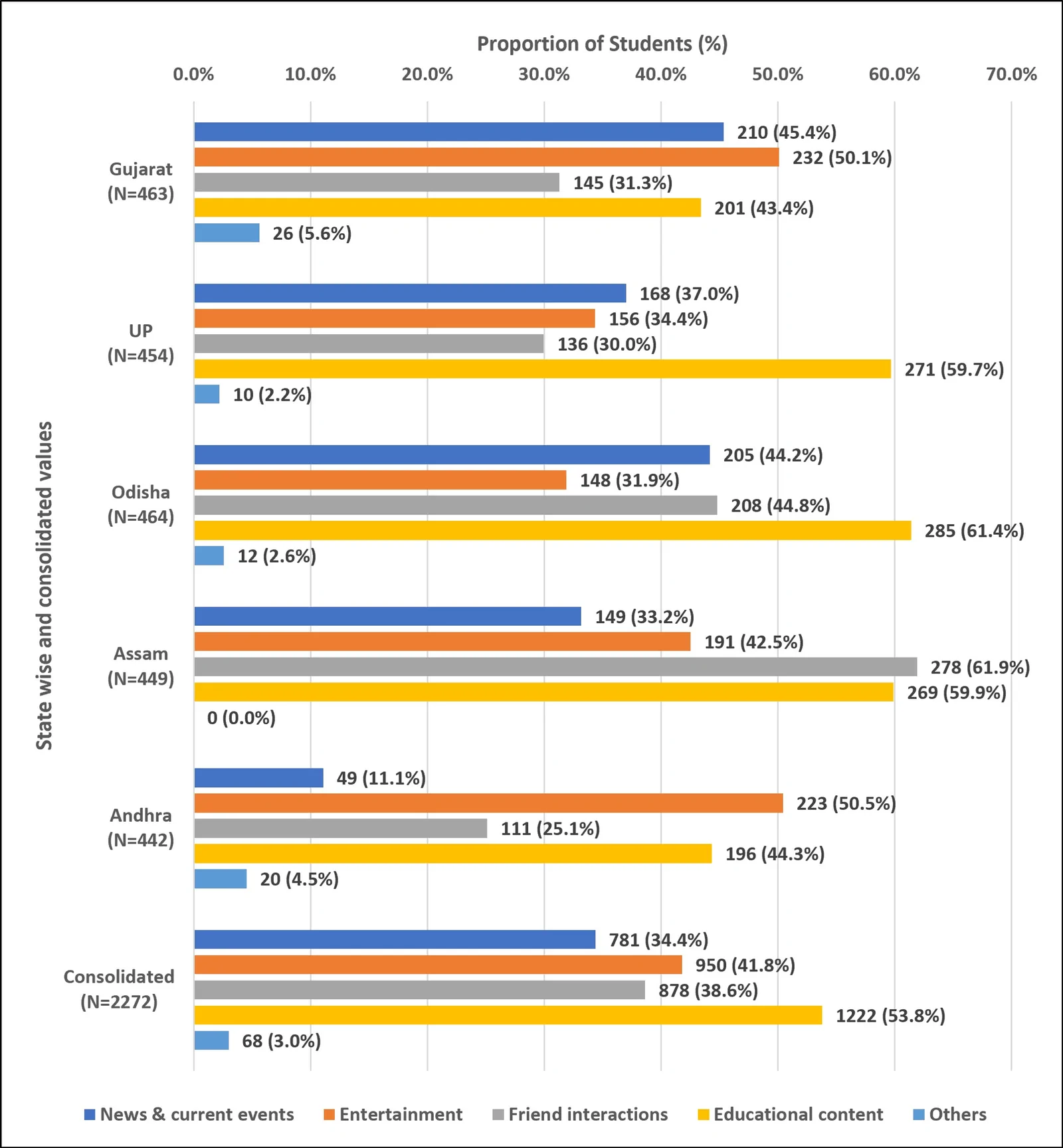
Type of content accessed by students on social media across selected states and consolidated sample.

Regarding health-related information sources, the majority of the respondents relied on social media (n=1411, 60.5%), of which WhatsApp, Facebook (Meta Platforms, Inc.), and Instagram were found to be the most used social media platforms. Newspapers/News channels were the second most-used health information sources among 1364 (58.5%) students (Figure 3). Notably, 1880 (80.6%) students believed that the health information available on social media was inaccurate or were unsure about the accuracy of this information, while only 451 (19.3%) considered it accurate (Table 5). However, approximately 1065 (46.9%) students still shared information with others occasionally, with 281 (12.4%) sharing it frequently (Table 6). Half of the students (n=1215, 53.5%) reported encountering health-related misinformation on social media (Figure 4). Additionally, most students were unable to distinguish between misinformation and disinformation (Tables 7, 8), with only 1225 (52.6%) being aware of reliable sources for health information (Figure 5). Remarkably, a mere 149 (6.4%) students reported receiving any training on identifying fake news, misinformation, or disinformation (Figure 6).

**Figure 3:**
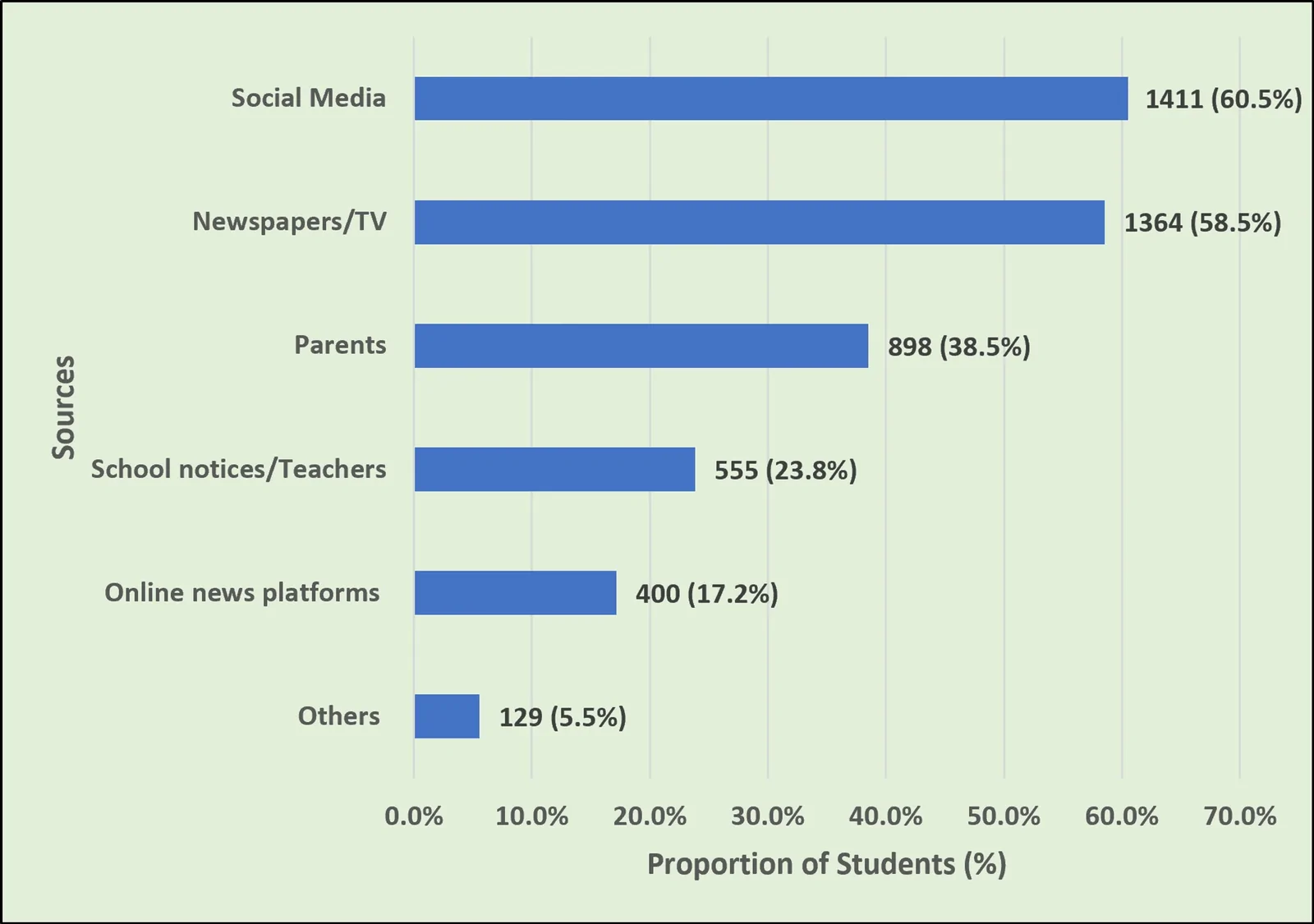
Distribution of sources from which students had accessed health-related news (N=2331)

**Table 5:** Students’ perception of the accuracy and reliability of health information shared on social media

State	Total participants	Yes, n (%)	No, n (%)	Unsure, n (%)
Gujarat	476	68 (14.3%)	235 (49.4%)	173 (36.3%)
Uttar Pradesh	458	91 (19.9%)	138 (30.1%)	229 (50.0%)
Odisha	468	179 (38.2%)	102 (21.8%)	187 (40.0%)
Assam	469	50 (10.7%)	232 (49.5%)	187 (39.9%)
Andhra Pradesh	460	63 (13.7%)	44 (9.6%)	353 (76.7%)
Consolidated	2331	451 (19.3%)	751 (32.2%)	1129 (48.4%)

**Table 6:** Distribution of students according to the frequency of sharing health information with others

State	Students with internet & social media access	Frequently, n (%)	Occasionally, n (%)	Rarely, n (%)	Never, n (%)
Gujarat	463	57 (12.3%)	253 (54.6%)	108 (23.3%)	45 (9.7%)
Uttar Pradesh	454	84 (18.5%)	215 (47.4%)	91 (20.0%)	64 (14.1%)
Odisha	464	61 (13.1%)	245 (52.8%)	109 (23.5%)	49 (10.6%)
Assam	449	61 (13.6%)	208 (46.3%)	93 (20.7%)	87 (19.4%)
Andhra Pradesh	442	18 (4.1%)	144 (32.6%)	72 (16.3%)	208 (47.1%)
Consolidated	2272	281 (12.4%)	1065 (46.9%)	473 (20.8%)	453 (19.9%)

**Figure 4:**
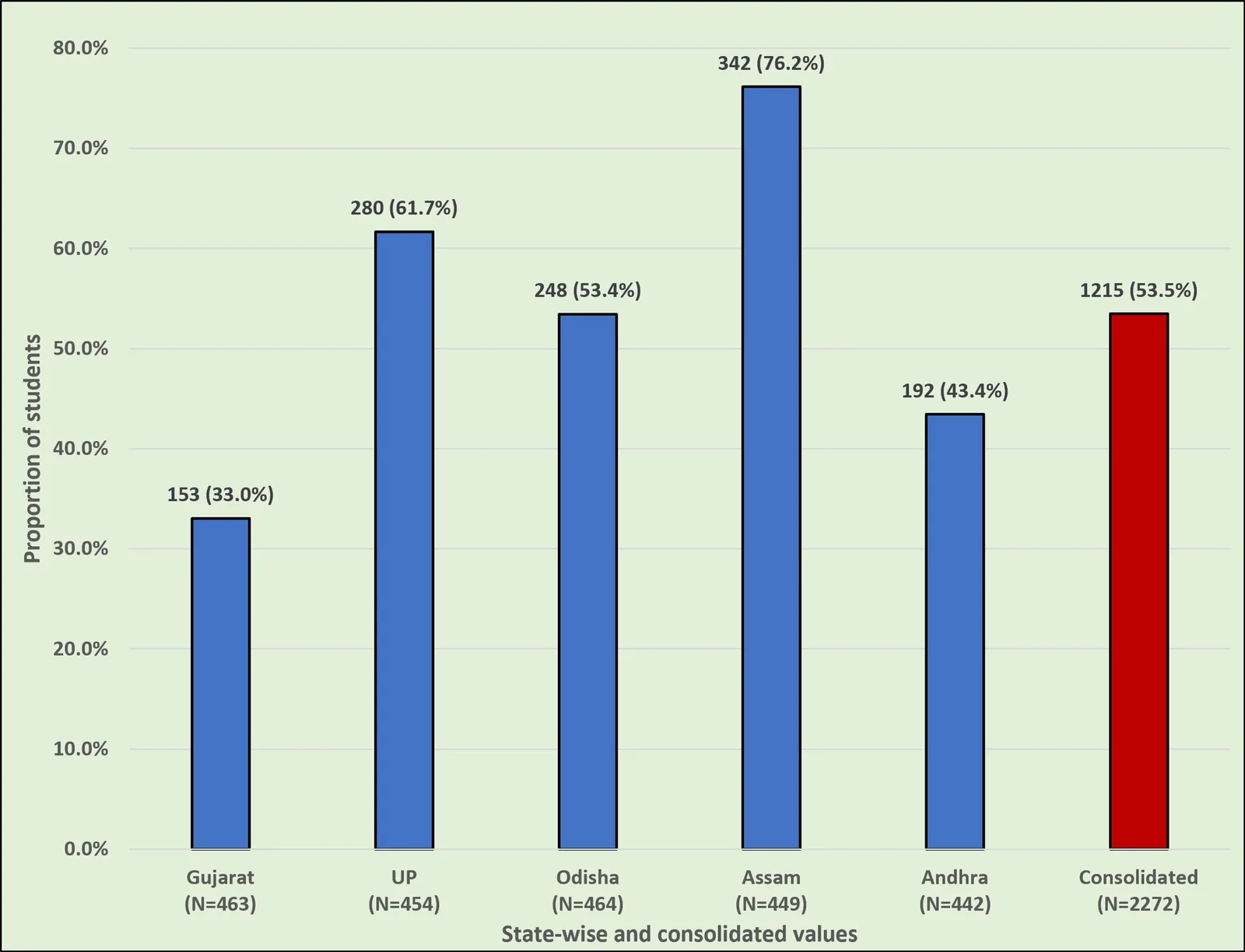
Proportion of students encountering health-related misinformation on social media and those reporting such content

**Table 7:** State-wise comparison of students’ understanding of misinformation Note: Participants could select more than one response option; therefore, the percentages may sum to more than 100%.

State	Total participants	Fake/False Information, n (%)	Harmful Information, n (%)	Unverified Information, n (%)	All of the Above, n (%)	None of the Above, n (%)
Gujarat	476	254 (53.4%)	105 (22.1%)	80 (16.8%)	129 (27.1%)	29 (6.1%)
Uttar Pradesh	458	156 (34.1%)	63 (13.8%)	92 (20.1%)	133 (29.0%)	46 (10.0%)
Odisha	468	231 (49.4%)	72 (15.4%)	93 (19.9%)	122 (26.1%)	18 (3.8%)
Assam	469	191 (40.7%)	70 (14.9%)	134 (28.6%)	207 (44.1%)	0 (0.0%)
Andhra Pradesh	460	261 (56.7%)	28 (6.1%)	66 (14.3%)	101 (22.0%)	31 (6.7%)
Consolidated	2331	1093 (46.9%)	338 (14.5%)	465 (19.9%)	692 (29.7%)	124 (5.3%)

**Table 8:** State-wise comparison of students’ understanding of disinformation Note: Participants could select more than one response option; therefore, the percentages may sum to more than 100%.

State	Total participants	Harmful Information, n (%)	Biased Information, n (%)	Unscientific Information, n (%)	Fake Information, n (%)	All of the Above, n (%)	None of the Above, n (%)
Gujarat	476	124 (26.1%)	47 (9.9%)	89 (18.7%)	216 (45.4%)	119 (25.0%)	40 (8.4%)
Uttar Pradesh	458	59 (12.9%)	55 (12.0%)	89 (19.4%)	128 (27.9%)	97 (21.2%)	57 (12.4%)
Odisha	468	150 (32.1%)	68 (14.5%)	123 (26.3%)	186 (39.7%)	36 (7.7%)	8 (1.7%)
Assam	469	106 (22.6%)	83 (17.7%)	165 (35.2%)	197 (42.0%)	97 (20.7%)	21 (4.5%)
Andhra Pradesh	460	68 (14.8%)	37 (8.0%)	102 (22.2%)	212 (46.1%)	47 (10.2%)	21 (4.6%)
Consolidated	2331	507 (21.8%)	290 (12.4%)	568 (24.4%)	939 (40.3%)	396 (17.0%)	147 (6.3%)

**Figure 5:**
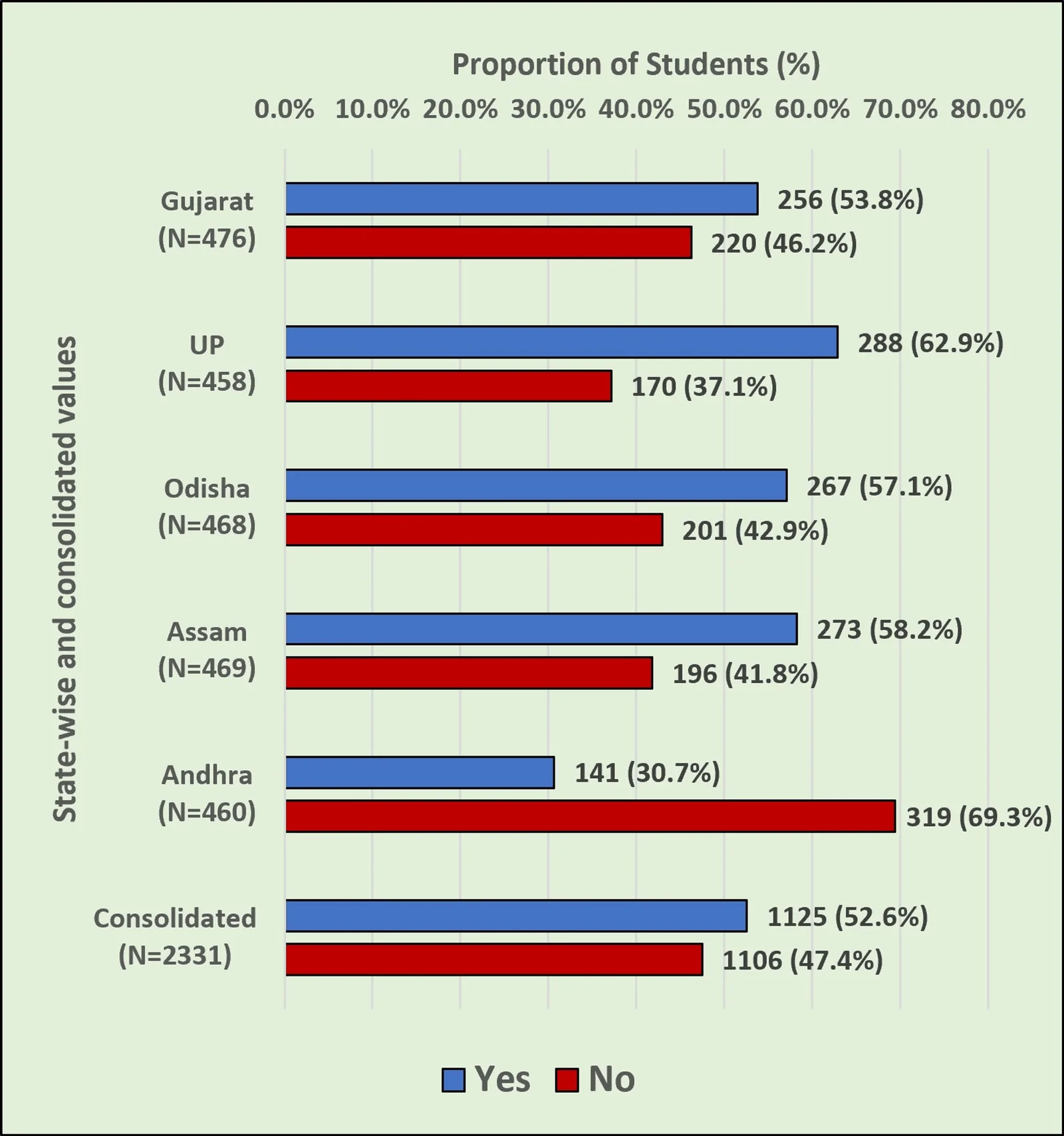
Proportion of students reporting awareness of reliable sources for health information, disaggregated by state UP: Uttar Pradesh

**Figure 6:**
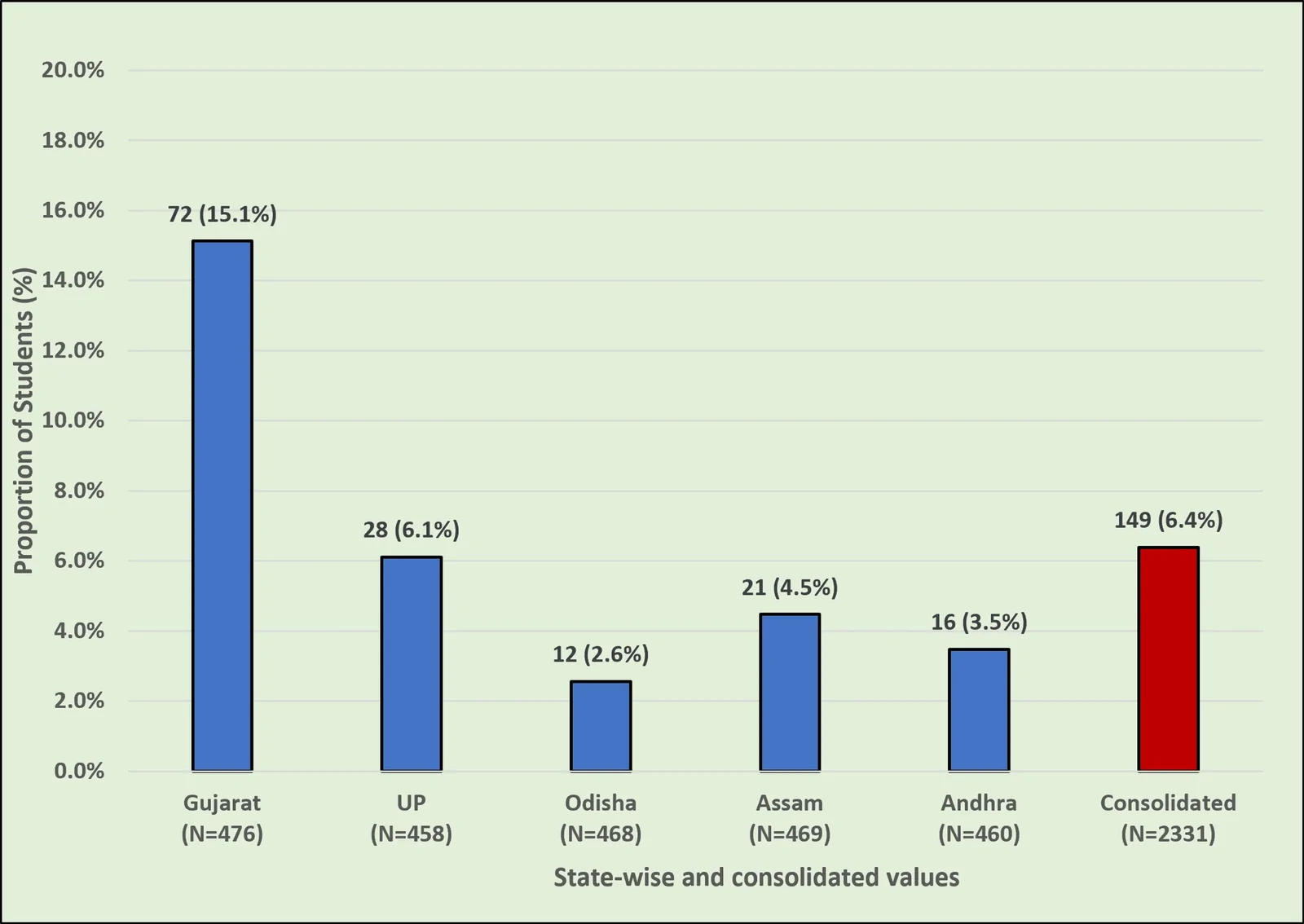
Proportion of students previously trained on identifying fake news, misinformation, or disinformation. UP: Uttar Pradesh

Outcomes of FGD with the students

The FGD revealed that most students actively used social media platforms like WhatsApp, Instagram, Snapchat (Snap Inc., Santa Monica, California, United States), YouTube, Telegram (Telegram FZ-LLC, Dubai, United Arab Emirates), Moj (Mohalla Tech Pvt Ltd, Bengaluru, Karnataka, India), Discord (Discord Inc., San Francisco, California, United States), and others, often spending around two hours daily (Table 9). In Gujarat, students were found to access social media 5-20 times a day. Some encountered false information, including unverified COVID-19 remedies. Common health topics discussed included nutrition, skincare and beauty products, fitness, diseases like dengue and COVID-19, seasonal flu remedies, and non-communicable diseases including diabetes, hypertension, mental health issues, and others. Students cited various sources for health information, with some relying on the Health Department, WHO, and news channels. In Assam, the Co-WIN App (https://www.cowin.gov.in/home) was valued for vaccination information. In UP, students faced challenges in navigating the reliable online health platform and used Google (Google LLC, Mountain View, California, India) for links to trusted sources. Students expressed the need for training on responsible social media use and standard operating procedures (SOP)/guidelines for circulating information. They also suggested developing Information, education, and communication (IEC) materials, organizing events, and competitions at the school level to combat misinformation and promote responsible social media use.

**Table 9:** Summary of key findings from FGDs with students (N = 1,972) FGD: focus group discussion; IEC: information, education, and communication; SOP: standard  operating procedure; WHO: World Health Organization; MoHFW: Ministry of Health and Family Welfare

Theme	Key Findings
Social media usage	Most students actively used social media platforms such as WhatsApp, Instagram, Snapchat, YouTube, Telegram, Moj, Discord, Facebook and others. Average use was approximately 1–2 hours per day, although some students reported up to 5 hours daily. In Gujarat, students reported accessing social media 5–20 times per day.
Health information accessed online	Students commonly encountered information related to nutrition, fitness, dieting, weight management, skincare and beauty, menstrual and personal hygiene, communicable diseases (COVID-19, Dengue, Malaria, seasonal flu) and non-communicable diseases such as diabetes, hypertension, obesity, cancer, heart diseases and mental health issues.
COVID-19 information experience	Students frequently obtained COVID-19 information through government websites, WHO, social media platforms, school WhatsApp groups and health-related online channels. Some reported exposure to misinformation and panic-inducing content during the pandemic.
Exposure to misinformation	Several students reported encountering fake information online, including unverified COVID-19 remedies, misinformation regarding vaccines and vaccine side effects, false celebrity news, misleading health claims, fake examination results, lottery scams and fraudulent banking messages.
Impact of misinformation	Misinformation caused confusion, stress, anxiety and panic among students and their families, particularly during the COVID-19 pandemic. Some students reported initially believing misleading health claims before verifying information with parents, teachers or trusted sources.
Trusted sources of health information	Students identified the Ministry of Health and Family Welfare (MoHFW), WHO, ICMR, UNICEF, government health departments, national news channels, newspapers and other official health platforms as reliable sources of information.
Verification practices and challenges	Students often cross-checked information using Google searches, official websites, teachers and parents. However, many reported difficulties distinguishing authentic from fake information because of the large volume of online content. In Uttar Pradesh, students noted that some health platforms were not user-friendly and often relied on Google links to access trusted sources.
State-specific observations	In Assam, students highlighted the usefulness of the Co-WIN app and MoHFW website for vaccination-related information and locating vaccination centres. In Uttar Pradesh, students reported challenges navigating reliable online health platforms.
Recommendations to combat misinformation	Students recommended training on responsible social media use, development of SOPs/guidelines for information screening before circulation, stronger monitoring systems, awareness campaigns, penalties for misinformation and promotion of source attribution. They also suggested developing IEC materials and organizing school-level events and competitions to improve media literacy and combat misinformation.

Outcomes of the needs assessment of the educators

It was found that all educators (100%) across all states had access to internet-enabled devices at home (Figure 7). A total of 269 educators (72.1%) reported using social media multiple times per day (Table 10), with WhatsApp (n=363, 97.3%), YouTube (n=332, 89.0%), and Facebook (n=247, 66.2%) being the most frequently used platforms (Figure 8). Educational content (n=302, 81.0%) and news (n=282, 75.6%) were the most accessed types of content overall (Table 11). A total of 187 (50.1%) educators frequently saw health information on social media, yet only 60 (16.1%) believed that health news on social media was accurate (Table 12). Notably, about 189 (50.7%) educators felt that all the information available on social media was not accurate. However, more than half the educators (n=205, 55.0%) admitted to sharing health information with others on social media. Of the 205 educators who shared health information via social media, only 89 (43.4%) reported that they frequently validated health news before sharing, with state-wise variation ranging from a high of 50.9% in Andhra Pradesh to a low of 25.0% in Assam. Only 82 (22.0%) educators reported feeling very confident about identifying false news. Additionally, only 82 (22.0%) ever reported any misleading or fake information on social media, with the highest being in Gujarat (31.5%) and the lowest in Assam (13.3%) (Table 12). Their understanding of misinformation included terms like wrong information, confusion, fake news, unscientific content, unauthorized information, and harmful content. Disinformation was perceived as unreliable, purposeless, incorrect, misleading, inappropriate, and invalid information. Most educators were unclear about the difference between misinformation and disinformation.

**Figure 7:**
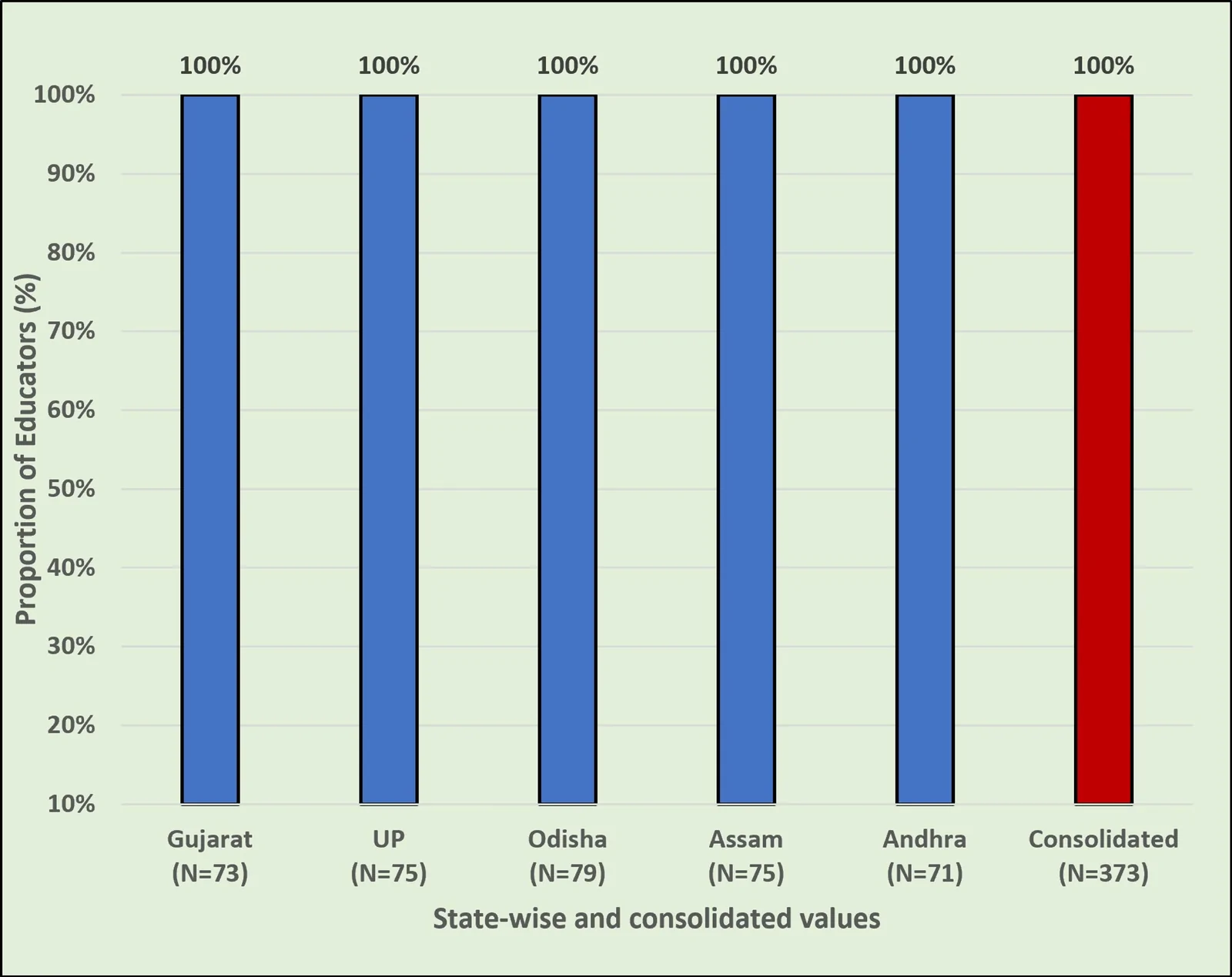
State-wise distribution of educators with access to internet-enabled devices

**Table 10:** Frequency of social media usage among educators across selected states and consolidated sample

States	Multiple times per day, n (%)	Once per day, n (%)	A few times per week, n (%)	Rarely or never, n (%)
Gujarat (N=73)	57 (78.1%)	11 (15.1%)	4 (5.5%)	1 (1.4%)
Uttar Pradesh (N=75)	50 (66.7%)	17 (22.7%)	8 (10.7%)	0 (0.0%)
Odisha (N=79)	62 (78.5%)	14 (17.7%)	3 (3.8%)	0 (0.0%)
Assam (N=75)	65 (86.7%)	4 (5.3%)	6 (8.0%)	0 (0.0%)
Andhra Pradesh (N=71)	35 (49.3%)	23 (32.4%)	4 (5.6%)	9 (12.7%)
Consolidated (N=373)	269 (72.1%)	69 (18.5%)	25 (6.7%)	10 (2.7%)

**Figure 8:**
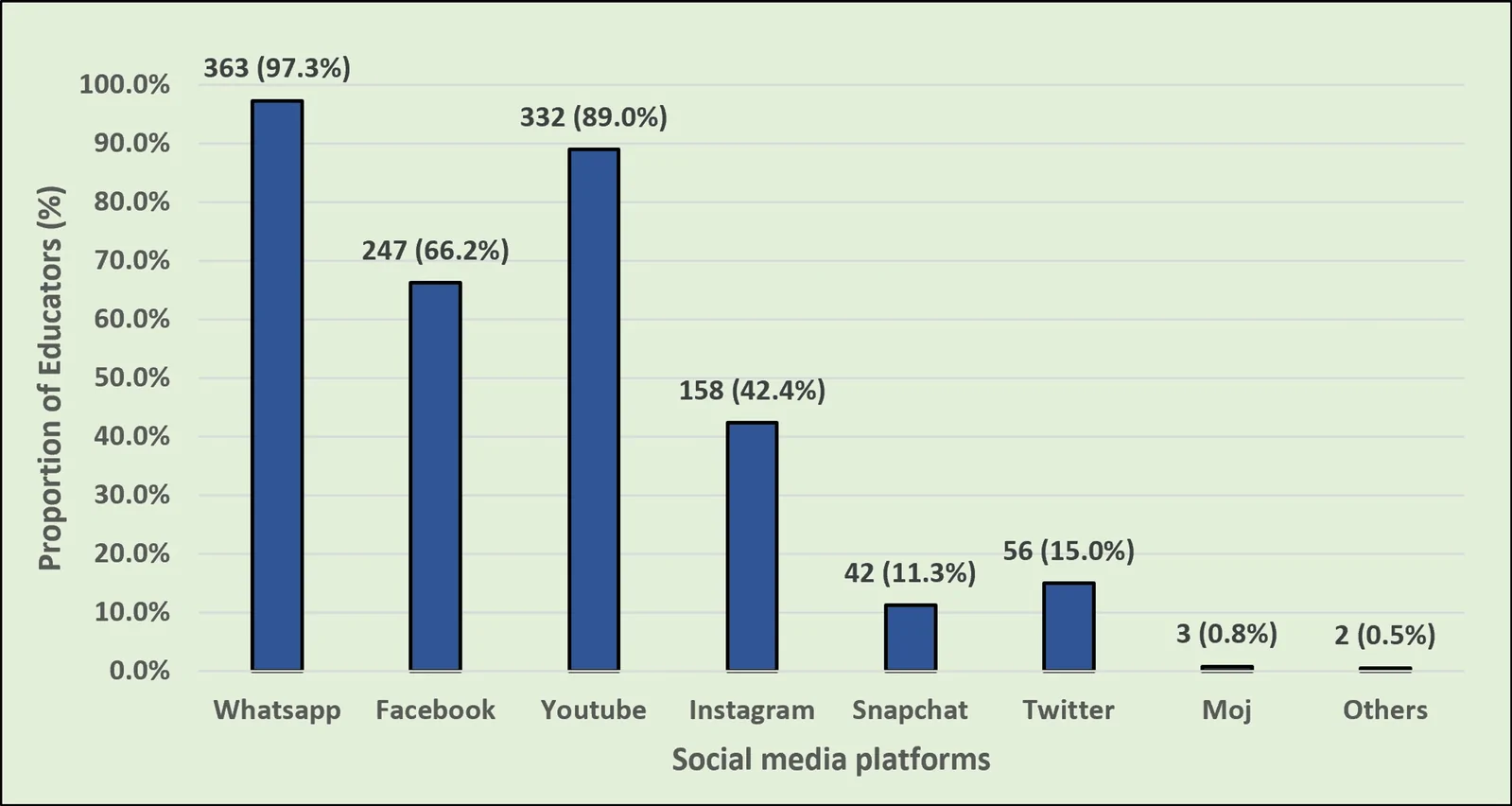
Distribution of social media platform usage among educators (N=373) Note: Participants could select more than one response option; therefore, the percentages may sum to more than 100%.

**Table 11:** Type of content accessed by educators on social media across selected states and consolidated sample Note: Participants could select more than one response option; therefore, the percentages may sum to more than 100%.

State	News & Current Events, n (%)	Entertainment, n (%)	Friend Interactions, n (%)	Educational Content, n (%)	Others, n (%)
Gujarat (N=73)	52 (71.2%)	22 (30.1%)	29 (39.7%)	52 (71.2%)	7 (9.6%)
Uttar Pradesh (N=75)	48 (64.0%)	18 (24.0%)	16 (21.3%)	48 (64.0%)	1 (1.3%)
Odisha (N=79)	66 (83.5%)	22 (27.8%)	46 (58.2%)	69 (87.3%)	1 (1.3%)
Assam (N=75)	73 (97.3%)	35 (46.7%)	55 (73.3%)	68 (90.7%)	4 (5.3%)
Andhra Pradesh (N=71)	43 (60.6%)	23 (32.4%)	14 (19.7%)	65 (91.5%)	6 (8.4%)
Consolidated (N=373)	282 (75.6%)	120 (32.2%)	160 (42.9%)	302 (81.0%)	19 (5.1%)

**Table 12:** State-wise comparison of teachers' exposure, perception, confidence, and practices related to health information and misinformation on social media

Indicators	Gujarat (n=73), n (%)	Uttar Pradesh (n=75), n (%)	Odisha (n=79), n (%)	Assam (n=75), n (%)	Andhra Pradesh (n=71), n (%)	Overall (N=373), n (%)
Exposure (Frequency of seeing health information on social media)						
Frequently	35 (47.9%)	44 (58.7%)	42 (53.2%)	37 (49.3%)	29 (40.8%)	187 (50.1%)
Occasionally	27 (37.0%)	22 (29.3%)	35 (44.3%)	35 (46.7%)	24 (33.8%)	143 (38.3%)
Rarely	11(15.1%)	9 (12.0%)	2 (2.5%)	3 (4.0%)	18 (25.4%)	41 (11%)
Perception (Accuracy of health news on social media)						
Accurate	3(4.1%)	10 (13.3%)	22 (27.8%)	0 (0.0%)	25 (35.2%)	60 (16.1%)
Not accurate	47 (64.4%)	40 (53.3%)	40 (50.6%)	39 (52.0%)	23 (32.4%)	189 (50.7%)
Unsure	23 (31.5%)	25 (33.3%)	17 (21.5%)	36 (48.0%)	23 (32.4%)	124 (33.2%)
Confidence in identifying false news/information						
Very confident	21 (28.8%)	20 (26.7%)	10 (12.7%)	14 (18.7%)	17 (23.9%)	82 (22.0%)
Somewhat confident	39 (53.4%)	45 (60.0%)	58 (73.4%)	50 (66.7%)	26 (36.6%)	218 (58.4%)
Hardly confident	9 (12.3%)	8 (10.7%)	6 (7.6%)	9 (12.0%)	25 (35.2%)	57 (15.3%)
Not confident	4 (5.5%)	2 (2.7%)	5 (6.3%)	2 (2.7%)	3 (4.3%)	16 (4.3%)
Practice of sharing health information						
Sharing health information on social media	37 (50.7%)	36 (48.0%)	51 (64.6%)	24 (32.0%)	57 (80.3%)	205 (55.0%)
Practice of validating health information before sharing						
	n=37	n=36	n=51	n=24	n=57	N=205
Frequently	17 (45.9%)	15(41.7%)	22(43.1%)	6(25.0%)	29 (50.9%)	89 (43.4%)
Occasionally	15(40.5%)	15(41.7%)	20(39.2%)	13(54.2%)	13(22.8%)	76 (37.1%)
Rarely	4(10.8%)	4(11.1%)	9(17.6%)	4(16.7%)	9(15.8%)	30 (14.6%)
Never	1(2.7%)	2(5.6%)	0 (0.0%)	1(4.2%)	6(10.5%)	10 (4.9%)
Practice of reporting fake/misleading information						
Reporting misleading/fake news or information	23 (31.5%)	11 (14.7%)	20 (25.3%)	10 (13.3%)	18 (25.4%)	82 (22.0%)

Outcome of the FGD with the educators

One educator FGD was conducted at each school, with approximately six educators participating in each FGD. Thus, a total of 450 educators participated in the FGDs across the five states and 75 schools. Most educators were actively engaged on social media platforms like WhatsApp, Facebook, YouTube, and Instagram, averaging two hours per day, with some spending five to six hours daily (Table 13). In Gujarat, teachers reported using social media 10-15 times per day due to connections with fellow educators and the government District Education Officer (DEO) office on WhatsApp groups. Health topics predominantly searched by educators include non-communicable diseases like hypertension, obesity, diabetes, cancer, heart diseases, COVID-19, reproductive and sexual health, and global warming, as well as diet, nutrition, exercise, yoga, and skin and beauty remedies. Other concerns include water-borne diseases, de-worming, eye infections, seasonal flu, dengue, malaria, tuberculosis, HIV, sexually transmitted infections (STIs), anaemia, and sickle cell anaemia. During the pandemic, educators played a crucial role in sharing COVID-19 updates, government guidelines, SOPs, preventive measures, mask availability, social distancing protocols, and vaccination information, primarily through WhatsApp groups.

**Table 13:** Summary of focus group discussion (FGD) findings among teachers MoHFW: Ministry of Health and Family Welfare (India); WHO: World Health Organization; UNICEF: United Nations Children's Fund; ICMR: Indian Council of Medical Research; AIIMS: All India Institute of Medical Sciences; RMRC: Regional Medical Research Centre; IEC: information, education, and communication; PTA: parent-teacher association; SMS: short message service

Theme	Key Findings	State-Specific Observations
Social media use	All teachers reported regular use of social media, often multiple times daily. Commonly used platforms included WhatsApp, Facebook, Instagram, YouTube, Google, InShorts, and Twitter. Most spent 1–2 hours daily, although some reported 6–7 hours per day.	Teachers in Gujarat reported frequent use of WhatsApp groups for communication with colleagues and District Education Office (DEO) officials, accessing social media 10–15 times daily.
Health information accessed online	Teachers frequently accessed information on COVID-19, vaccination, hypertension, diabetes, obesity, cancer, mental health, reproductive and sexual health, tuberculosis, dengue, HIV/AIDS, nutrition, yoga, and healthy lifestyles through government websites, WHO, MoHFW, YouTube, Google, and news channels.	Teachers demonstrated high awareness of health information sources across all states.
Role during COVID-19 pandemic	Teachers actively disseminated COVID-19 information, government guidelines, preventive measures, vaccination messages, hygiene practices, and myth-busting information through WhatsApp groups, SMS, and online classes.	Odisha teachers highlighted school sanitization before reopening, while Assam teachers emphasized digital safety and privacy education.
Challenges in information dissemination	Major challenges included students’ limited access to digital platforms, contradictory online information, difficulty identifying authentic sources, and verifying information before dissemination.	Teachers in Uttar Pradesh reported significant challenges addressing vaccine hesitancy caused by misinformation.
Exposure to misinformation	Teachers commonly encountered misinformation related to COVID-19 transmission, treatment, home remedies, vaccine safety, oxygen shortages, and exaggerated case reports. Rumours often originated from social media platforms.	Uttar Pradesh teachers specifically reported myths such as onion consumption curing COVID-19, sunlight killing the virus, and vaccines causing fatal blood clots.
Impact of misinformation	Misinformation created fear, confusion, vaccine hesitancy, and anxiety among communities. Teachers reported verifying information through official government websites, WHO resources, and healthcare professionals before sharing.	Teachers perceived students as more vulnerable to misinformation due to limited fact-checking skills.
Challenges in identifying reliable sources	Many teachers found the abundance of online information confusing and reported difficulty distinguishing reliable from unreliable sources. Verification through WHO, government websites, and fact-checking tools was common practice.	Odisha teachers viewed multiple sources as useful but emphasized verification, while Assam teachers considered the abundance of information both a challenge and an opportunity.
Trusted health information sources	Frequently cited reliable sources included MoHFW, WHO, UNICEF, ICMR, AIIMS, RMRC, government websites, health helplines, newspapers, and national news channels.	Government health advisories were considered the most trustworthy sources across states.
Strategies for educating students	Teachers recommended regular awareness sessions, audiovisual materials, local-language IEC materials, classroom discussions, competitions, parent engagement, and digital literacy training to promote responsible social media use.	Odisha teachers suggested regular interactions with healthcare experts. Assam teachers emphasized parental involvement and positive parenting. Uttar Pradesh teachers recommended a dedicated government app for teachers and students.
Information-sharing networks	Most teachers participated in school, class, family, and professional WhatsApp groups. Information shared included academic updates, health information, fitness tips, and current affairs.	Gujarat teachers recommended a government-managed health information website for public verification. Uttar Pradesh teachers preferred informal offline discussions over online health groups.
Recommendations for combating misinformation	Teachers recommended school-level committees, peer educator programmes, teacher orientations, PTA engagement, IEC materials, stronger regulation of online platforms, and government-supported fact-checking mechanisms.	Andhra Pradesh teachers advocated strict action against websites spreading misinformation. Assam teachers suggested incorporating digital safety and cyber awareness into school curricula and limiting mobile phone use in schools.
Suggested role of teachers	Teachers highlighted the need to integrate responsible social media use into school curricula, conduct regular awareness programmes, engage parents, organize competitions, and provide counselling on digital literacy and responsible information sharing.	Consensus across states supported institutionalizing media and health literacy education within schools.

Challenges in sharing and understanding information included contradictory information across platforms, difficulty verifying online information, and the circulation of fake vaccination news causing confusion among the community, particularly in UP. False information encountered included claims that yoga and exercise could prevent COVID-19, information about vaccine side effects, unfounded reports of COVID-19 case increases, false home remedy claims, and misguided beliefs about alcohol preventing throat infections. Teachers identified false information by cross-referencing with local doctors and reputable sources like the Ministry of Health and Family Welfare (MoHFW), WHO, government sources, United Nations Children's Fund (UNICEF), ICMR, ICMR-Regional Medical Research Centres (RMRCs), All India Institute of Medical Sciences (AIIMS) websites, helplines, and national news channels and newspapers.

To engage children on responsible social media use regarding health news, teachers suggested verifying forwarded information, conducting regular interactive sessions, using audio-visual presentations, developing materials in local languages, raising awareness during parent-teacher association (PTA) meetings, and distributing brochures in local languages. Many educators participated in various WhatsApp groups, including school/class, family, and batchmates groups, sharing lesson details, school-related information, and health and fitness information. Some also shared information on Facebook and Instagram.

## Discussion

Social media has emerged as the main communication channel among all age groups. This study found nearly universal internet access among students and teachers, with high levels of exposure to social media platforms, making social media a prominent source of health information. A shift was observed in the most popular social media platforms among students compared to previous research. Instagram, WhatsApp, and YouTube emerged as the top choices, whereas earlier studies, such as the e-Safety study (2016) [6], highlighted YouTube and Facebook as dominant platforms.

Conversely, among educators, WhatsApp and Facebook remained the primary social media platforms, aligning with findings from other studies like the Gintux Report (2024) [7]. Interestingly, X (formerly Twitter; X Corp., Bastrop, Texas, United States) emerged as a significant platform among teachers, as indicated by the Social Media Habits of Teachers report (2023) [8], which surveyed over 5000 school-based educators in the United Kingdom using Teacher Tapp (Education Intelligence Limited, Poole, United Kingdom), highlighting the evolving landscape of social media usage among educators.

Examining the implications of relying on social media as a news source unveils challenges, including exposure to fake news disseminated by unreliable sources and susceptibility to consuming biased or filtered information. This study gauged the awareness of fake news prevalence online among students and teachers, revealing that only 32.2% of students and approximately 50.6% of teachers were aware of this issue. Furthermore, a significant proportion of the participants in the current study admitted to sharing the health news without verifying its authenticity. Thus, this study highlighted gaps in understanding among most students and teachers in identifying and validating health misinformation and disinformation. This pattern echoes findings from McGrew et al. (2018) [9], who found that students primarily relied on the information and images contained within social media posts to make judgments about accuracy, source, and reliability, without verifying accuracy or authenticity beyond the content of the posts.

The results of this study highlight the growing need for social media literacy by providing empirical evidence on specific concerns faced by teachers and students in navigating the complex digital world filled with a plethora of health information. During FGDs, both students and teachers expressed the need for imparting social media literacy as an essential skill to navigate evidence-based health information and understand false information. Evidence from previous studies, including those by Emilda [10], Dashtestani and Hojatpanah [11], Hassan and Mirza [12], and Bansal and Misra [13], points to a broader and consistent concern: while students are increasingly exposed to and engaged with digital and social media platforms, educators’ digital competencies and preparedness to critically evaluate and communicate online information may not be developing at the same pace. An important finding in the present study was the apparent knowledge-practice gap among educators. Despite substantial exposure to online health information and a relatively high level of skepticism regarding the accuracy of such information, 55% of educators reported sharing information without verification. This suggests that recognizing that online information may be inaccurate does not necessarily translate into consistent verification behaviour. The gap may reflect limited practical skills in evaluating sources, time constraints, reliance on familiar information sources, or the ease and immediacy of sharing content through social media platforms. These findings highlight the need for interventions that move beyond awareness and focus on practical skills for source verification, evidence appraisal, and responsible information sharing.

A study on health information-seeking behaviour by Gray et al. in the United Kingdom and the United States, involving 157 English-speaking students aged 11-19, revealed that the internet is a convenient and confidential source of health information [14]. In Germany, similar research with university students highlighted the need for tailored interventions to strengthen digital health literacy and improve the quality of health-related information online [15]. In India, a study in Jhalawar, Rajasthan, by Thakan et al. found that adolescents primarily sought nutrition information in formal settings like school but used the internet for personal needs, emphasizing the importance of guiding adolescents in responsible internet use [16]. A study in Texas by Kortum et al. revealed that much of the health information on the internet, especially regarding vaccines, is inaccurate, stressing the importance of critical evaluation skills [17]. In Jeddah, Saudi Arabia, Alghamdi et al. found that 92.7% of students frequently searched the internet for health information, with 84.8% believing it improved their health status, suggesting the internet is a valuable resource that influences health-related decisions [18].

Our findings, along with these national and international studies, highlight the critical need to address the digital literacy requirements of students and teachers and implement tailored strategies to enhance their online health-information-seeking behaviour and promote responsible and safe use of social media. A major part of this is to ensure that the official information is timely, evidence-based, and accessible, complementing skill-building on health-seeking behaviours. This needs to be considered for a holistic problem-solving approach. The trend shows that students are increasingly reliant on digital platforms for health-related information, necessitating targeted interventions that cater to their specific capacities and preferences. These interventions should educate, engage, and empower students to navigate the complexities of social media effectively, responsibly, and safely. 

Limitations of the study and future recommendations

This study has several limitations that should be considered when interpreting the findings.

First, the cross-sectional design provides a snapshot of knowledge, perceptions, and self-reported practices at a single point in time and therefore does not permit the establishment of causal relationships between exposure to online health information, digital literacy, and related behaviours. Second, the study relied primarily on self-reported data from students and educators, which may be affected by recall and social desirability biases. Participants may have overreported desirable practices, such as verifying information before sharing, or underreported less desirable behaviours. Although the qualitative component provided additional contextual insights, it cannot completely eliminate these potential biases. Third, school self-selection may have introduced selection bias. Participating schools were identified and selected through the respective state/district departments of education, and schools that declined participation or were unavailable may have differed systematically from participating schools in terms of infrastructure, internet connectivity, availability of digital resources, or characteristics of students and educators. Such differences may have influenced the observed estimates, including the relatively high levels of reported internet and social media access. Fourth, although the study included schools from five geographically diverse states, it should not be interpreted as a nationwide assessment. The findings provide insights from selected settings representing different regions of India; however, their generalizability to all states, union territories, and school populations should be interpreted cautiously. The purposive selection of states and the selection of participating schools through education authorities further limit the extent to which the findings can be extrapolated to the wider Indian school population.

Fifth, the use of closed-ended questions to assess perceptions, confidence, and understanding may not have fully captured the depth and complexity of participants’ views regarding misinformation and disinformation. The qualitative component helped address this limitation by allowing participants to elaborate on their experiences and perspectives; however, the depth of individual responses may have been constrained by the relatively large size of some student focus groups and the time available within school settings. Sixth, the data collection tools were adapted for use across different settings and languages. Although the tools were reviewed for linguistic and contextual appropriateness, formal back-translation and systematic validation of all translated versions were not undertaken/documented. Consequently, subtle differences in interpretation across language versions cannot be completely excluded. Seventh, state-wise disaggregated data were not available for all indicators, particularly those related to perceptions and understanding. This limited detailed comparisons across states and restricted the ability to explore geographical variation in greater depth. Eighth, the analysis was primarily descriptive, and inferential statistical tests were not undertaken to assess the statistical significance of observed differences between groups. Accordingly, comparisons between students and educators or across other subgroups should be interpreted as descriptive rather than as evidence of statistically significant differences.

Ninth, contextual factors such as differences in digital infrastructure, internet availability, media exposure, and school-level environments were not examined in detail. These factors may influence participants’ access to, use of, and engagement with online health information and may partly explain differences in reported behaviours. Tenth, the study assessed perceptions and self-reported practices rather than the objective accuracy of health information accessed, evaluated, or shared by participants. Consequently, the study cannot determine the extent to which the information participants encountered or disseminated was factually accurate, misleading, or false. Finally, the limited time available for data collection within schools may have constrained the depth of both quantitative and qualitative assessment. In particular, the relatively large number of participants in some focus group discussions, necessitated by the number of eligible students and practical time constraints, may have limited opportunities for detailed individual contributions.

Future studies should consider longitudinal designs, broader and more representative sampling, validated multilingual instruments, and objective assessment of digital and health-information literacy and the accuracy of information accessed or shared. Such approaches would provide a more comprehensive understanding of misinformation and health-information-seeking dynamics among school students and educators.

## Conclusions

This study highlights the need to strengthen digital and health information literacy among school students and educators, particularly their ability to critically evaluate the credibility of online health information before using or sharing it. Integrating age-appropriate digital/media literacy modules into school curricula, alongside targeted training for educators, could strengthen responsible online health-information practices and digital citizenship. Such measures may contribute to reducing the spread of health misinformation and promoting safer, more informed use of digital platforms within school communities.

## Appendices

Appendix A: student questionnaire

**Table 14:** Student Questionnaire for Assessing Knowledge of Students on Social Media Use and Access to Online Health Information

Section A. Participant Information and Informed Assent			
Item		Description	
Study Title		Assessing the Knowledge of Students on Social Media Use and Access to Online Health Information	
Purpose		To assess students' use of social media, health information-seeking behaviour, awareness of misinformation/disinformation, and digital literacy.	
Participation		Voluntary. Students may withdraw at any time without affecting their academic standing.	
Estimated Time		10–15 minutes	
Confidentiality		Responses are anonymous and used only for research purposes. No personal identifiers will be collected.	
Permission		Permission has been obtained from the school authorities. Student assent is required before participation.	
Consent		□ I Agree □ I Do Not Agree	
Section B: Questionnaire			
Q. No.	Domain	Question	Response Options
1	Internet Access	Do you have access to an internet-enabled device at home (mobile phone, tablet, laptop, or computer)?	Yes / No
2	Personal Device	Do you own or have access to your own internet-enabled device?	Yes / No
3	Social Media Awareness	Have you heard of social media platforms such as Facebook, Instagram, Twitter, or Snapchat?	Yes / No
4	Social Media Platforms Used	Which social media applications do you use? (Select all that apply)	WhatsApp; Facebook; YouTube; Instagram; Snapchat; Twitter (X); Moj; Koo; Other (specify)
5	Preferred Platform	Which social media platform do you use the most?	WhatsApp; Facebook; YouTube; Instagram; Snapchat; Twitter(X); Moj; Koo; Other (specify)
6	Frequency of Use	How often do you access social media?	Multiple times/day; Once/day; A few times/week; Rarely/Never
7	Content Accessed	What type of information do you usually access on social media?	News/current events; Entertainment; Friend interactions; Educational content; Other (specify)
8	Health Information Seeking	Do you read health news (including COVID-19) online?	Yes / No
9	Frequency of Reading Health News	How often do you read online health news?	Frequently; Sometimes; Rarely; Never
10	Sources of Health Information	Where do you usually obtain health information? (Multiple responses allowed)	Facebook/Instagram; Twitter; WhatsApp; Online news portals; Newspapers/TV; School notices; Parents; Other (specify)
11	Exposure to Health News	How often do you encounter health information on social media?	Frequently; Occasionally; Rarely; Never
12	Perceived Accuracy	Do you believe health information shared on social media is always accurate and reliable?	Yes; No; Unsure
13	Sharing Behaviour	Have you ever shared health information on social media?	Yes / No
14	Frequency of Sharing	How often do you share health information with friends or family?	Frequently; Sometimes; Rarely; Never
15	Fact Checking	How often do you verify health information before sharing it?	Frequently; Sometimes; Rarely; Never
16	Exposure to Misinformation	Have you encountered health-related misinformation on social media?	Yes / No
17	Perceived False Information	How often do you find online health information to be misleading or false?	Frequently; Sometimes; Rarely; Never
18	Awareness of Reliable Sources	Are you aware of reliable organizations that provide accurate health information?	Yes / No
19	Verification Sources	If yes, which sources do you use to verify health information?	Open-ended response
20	Understanding of Misinformation	What do you understand by the term misinformation? (Multiple responses allowed)	Fake/false information; Harmful information; Unverified information; All of the above; None of the above
21	Understanding of Disinformation	What do you understand by the term disinformation? (Multiple responses allowed)	Harmful information; Biased information; Unscientific information; Fake information; All of the above; None of the above
22	Previous Training	Have you attended any training/workshop/lecture on fake news or misinformation?	Yes / No
22a	Training Details	If yes, provide the name, date, and topics covered.	Open-ended response
23	Reporting Behaviour	Have you ever reported misleading or fake information?	Yes / No
23a	Reporting Details	If yes, where did you report it?	Open-ended response

Appendix B:  educator questionnaire

**Table 15:** Educator Questionnaire for Assessing Educators' Knowledge on Social Media Use and Access to Online Health Information

Section A. Participant Information and Informed Consent			
Item		Description	
Study Title		Assessing Educators' Knowledge on Social Media Use and Access to Online Health Information	
Purpose		To assess educators' use of social media, health information-seeking behaviour, awareness of misinformation/disinformation, and digital literacy.	
Participation		Voluntary. Participants may withdraw at any time without any consequences.	
Estimated Time		10–15 minutes	
Confidentiality		Responses are anonymous, confidential, and used only for research purposes. No personal identifiers will be collected.	
Consent		By participating, you confirm that you have read the study information and voluntarily agree to participate. □ I Agree □ I Do Not Agree	
Section B. Questionnaire			
Q. No.	Domain	Question	Response Options
1	Internet Access	Do you have access to an internet-enabled device at home (mobile phone, tablet, laptop, or computer)?	Yes / No
2	Personal Device	Do you have access to your own internet-enabled device?	Yes / No
3	Social Media Platforms Used	Which social media applications do you use? (Select all that apply)	WhatsApp; Facebook; YouTube; Instagram; Snapchat; Twitter (X); Moj; Koo; Other (specify)
4	Preferred Platform	Which social media platform do you use the most?	WhatsApp; Facebook; YouTube; Instagram; Snapchat; Twitter (X); Moj; Koo; Other (specify)
5	Frequency of Use	How often do you access social media?	Multiple times/day; Once/day; A few times/week; Rarely/Never
6	Content Accessed	What type of information do you usually access on social media?	News/current events; Entertainment; Friend interactions; Educational content; Other (specify)
7	Health Information Seeking	Do you read health news (including COVID-19) online?	Yes / No
8	Frequency of Reading Health News	How often do you read health news online?	Frequently; Sometimes; Rarely; Never
9	Sources of Health Information	Which platforms do you use to access health information? (Multiple responses allowed)	Twitter (X); Facebook; WhatsApp; Instagram; YouTube; Koo; Inshorts; 60 Seconds Now; Government websites; WHO website; Online newspapers; Online news channels; Other (specify)
10	Exposure to Health Information	How often do you encounter health information on social media?	Frequently; Occasionally; Rarely; Never
11	Perceived Accuracy	Do you believe that health information shared on social media is always accurate and reliable?	Yes; No; Unsure
12	Sharing Behaviour	Have you ever shared health information on social media?	Yes / No
13	Frequency of Sharing	How often do you share health information with students, friends, or family?	Frequently; Sometimes; Rarely; Never
14	Fact Checking	How often do you verify health information before sharing it?	Frequently; Sometimes; Rarely; Never
15	COVID-19 Information Sharing	During the COVID-19 pandemic, did you share COVID-19-related information with students?	Yes / No
16*	Frequency of COVID-19 Sharing	(If Yes to Q15) How frequently did you share COVID-19 information with students?	Frequently; Sometimes; Rarely; Never
17*	Channels Used	(If Yes to Q15; multiple responses allowed) Where did you share COVID-19 information?	WhatsApp; Other social media; School website/bulletin board; Email; SMS; Phone/video calls with parents
18*	Sources Used	(If Yes to Q15) From which sources did you obtain COVID-19 information before sharing it?	Open-ended response
19*	Verification Practice	(If Yes to Q15) Did you verify the accuracy of COVID-19 information before sharing it?	Yes / No
20	Verification Sources	Which sources do you most frequently use to verify health information before sharing it?	Open-ended response
21	Confidence in Identifying False Information	How confident are you in identifying false or misleading health information online?	Very confident; Somewhat confident; Hardly confident; Not confident
22	Understanding of Misinformation	What does the term misinformation mean to you?	Open-ended response
23	Understanding of Disinformation	What does the term disinformationmean to you?	Open-ended response
24	Previous Training	Have you attended any training, workshop, or lecture on fake news or misinformation?	Yes / No
24a	Training Details	If yes, provide the name, date, and topics covered.	Open-ended response
25	Reporting Behaviour	Have you ever reported misleading or fake information?	Yes / No
25a	Reporting Details	If yes, where did you report it?	Open-ended response
26	Current Practice	Do you continue to share health-related information with students?	Yes / No
*Conditional questions: Questions 16–19 were administered only to respondents who answered "Yes" to Question 15.			

Appendix C:  FGD guide for students

**Table 16:** Focus Group Discussion Guide for Students

A. Focus Group Information		
Item		Details
Participants		≥30 students (Grades 9–12) per school
Duration		25–30 minutes
Method		Audio-recorded Focus Group Discussion
Facilitators		Project team member(s) with one teacher present
Consent		Written informed assent obtained before participation
B. Administrative Details		
Field		Details
State/City		__________
School		__________
Number of Students		__________
Date		__________
C. Discussion Guide		
Q. No.	Theme	Discussion Question
1	Social media use	Which social media platforms do you use most frequently, and how much time do you spend on them each day?
2	Health information	What types of health-related information do you commonly access on social media?
3	COVID-19 experience	What was your experience accessing COVID-19 information online during the pandemic?
4	Misinformation	Have you encountered false or misleading information online? Please describe your experience.
5	Impact	How has online misinformation affected you or others?
6	Trusted sources	Which organizations or websites do you trust for accurate health information?
7	Verification	How do you verify whether health information online is reliable?
8	Challenges	What challenges do you face in identifying trustworthy online information?
9	Information sharing	Do you share health or news information with friends or classmates? If yes, through which platforms?
10	Recommendations	What recommendations would you suggest to reduce misinformation and improve digital health literacy?

Appendix  D: FGD guide for educators

**Table 17:** Focus Group Discussion Guide for Teachers

A. Focus Group Information		
Item		Details
Participants		≥5 teachers (Grades 9–12); Principals/Vice-Principals may participate
Duration		25–30 minutes
Method		Audio-recorded Focus Group Discussion
Consent		Written informed consent obtained before participation
B. Administrative Details		
Field		Details
State/City		__________
School		__________
Number of Participants		__________
Date		__________
Time		__________
C. Discussion Guide		
Q. No.	Theme	Discussion Question
1	Social media use	Which social media platforms do you use most frequently, and how much time do you spend on them each day?
2	Health information	What types of health-related information do you commonly encounter on social media?
3	COVID-19 communication	What role did you play in sharing COVID-19 information with students during the pandemic?
4	Communication challenges	What challenges did you face while sharing health or COVID-19 information with students?
5	Misinformation	Have you encountered false or misleading health information online? How did you recognize it?
6	Impact and response	How has misinformation affected you or your school community, and how did you address it?
7	Reliable information	How do you identify reliable health information sources online?
8	Student education	What strategies can schools use to educate students about responsible use of social media and health information?
9	Information sharing	Do you share health-related information with colleagues? If yes, through which platforms and sources?
10	Policy recommendations	What recommendations would you suggest for government guidelines to reduce misinformation in educational institutions?
11	Teachers' role	What role should teachers play in helping students become responsible consumers and sharers of online health information?
